# Implant-supported zirconia fixed partial dentures cantilevered in the lateral-posterior area: A 4-year clinical results

**DOI:** 10.34172/joddd.2022.041

**Published:** 2022-12-30

**Authors:** Giuseppe D'Albis, Vincenzo D'Albis, Bart Susca, Micol Palma, Nizar Al Krenawi

**Affiliations:** ^1^Periodontology and Implantology Department, University of Federico II of Naples, Naples, Italy; ^2^Department of Orthodontics, University of Rome “Tor Vergata”, Rome, Italy; ^3^Dental Technician, Mola di Bari (BA), Italy; ^4^Interdisciplinary Department of Medicine, University of Bari, Bari, Italy

**Keywords:** CAD-CAM, Cantilever, Dental implants, Digital workflow, Zirconia

## Abstract

**Background.:**

Implant-supported cantilever prostheses enable a more straightforward rehabilitation and may be a therapeutic option to reduce treatment morbidity, costs, and time. This study evaluated the clinical outcomes of fixed implant-supported partial dentures made of monolithic zirconia with a cantilever design to replace missing posterior teeth.

**Methods.:**

Fifteen partially edentulous patients received 34 implants and were provided with 16 zirconia fixed partial prostheses (FPPs) with one cantilever extension replacing mandibular or maxillary missing posterior and lateral teeth. Patients were re-examined for up to 4 years. Patient ages ranged from 41 to 65 years, with a mean age of 53±12 years; 47% were female, and 53% were male. The patients were observed for a mean period of 42±6 months with a minimum of 3 years and a maximum of 4 years.

**Results.:**

Peri-implantitis was observed in two cases. No chipping or fracture of any FPP was detected. Loosening of the abutment screw was a technical complication in one case. The rehabilitation survival rate was 100%. Implant-supported zirconia FPP with one mesial cantilever extension provides an aesthetic, functional treatment alternative to replace missing molars, premolars, and canines. These excellent clinical outcomes occurred over a mean observation time of 42±6 months.

**Conclusion.:**

Using monolithic zirconia milled with CAD-CAM technology might be an alternative to the metal-ceramic restoration in implant-supported FPP with one cantilever.

## Introduction

There appears to be a trend toward minimally invasive approaches in modern dentistry. Minimally invasive dentistry achieves treatment objectives using the least invasive surgical approach.^1^ The goal is to increase patient satisfaction, decrease postoperative discomfort and morbidity, and reduce treatment periods and costs.

Invasive interventions increase the risk of soft and hard tissue complications, including infection, swelling, and morbidity.^2^ It is critical to determine whether to perform reconstructive surgery after considering patients’ age, systemic health disorders, ability for oral hygiene, and cooperation with treatment. An alternative treatment strategy should be considered if invasive surgery is not suggested.^3^

Implants with a mesial cantilever fixed prosthesis are a treatment option that avoids pre-implant reconstructive or regenerative procedures in partially edentulous jaws.^4,5^ The long-term success of this treatment is well-documented. Several studies on single implant cantilevers have shown that this treatment option is predictable and can rehabilitate the two consecutive missing teeth in the posterior area.^6,7^

In studies involving this type of rehabilitation, the material of choice is metal-ceramic. Only one study has reported using zirconia as a prosthetic material, with no reported differences in survival rates concerning metal-ceramic restorations.^8^

The use of zirconia in cantilever restorations is widely discussed, and many authors believe that metal-ceramic remains the gold standard material.^9^ Concurrent with simplifications of protocols and the benefits of digitalization, zirconia in prosthetic restorations has become popular among digital prosthodontists. The benefits are significant in these procedures, leading to more predictable results, better prosthesis framework fit, and a reduction in realization time.^10,11^

The most salient problem with monolithic zirconia is cantilever fracture; nevertheless, there are few studies on the relationship between the length of the cantilever extension and framework thickness around the fracture. Some in vitro evaluations found a higher rate of load-to-fracture, with a proportion relation once the cantilevers were shorter, indicating that the cross-sectional connector area was large.^12-14^

 Considering the excellent results of the implant-supported partial cantilever prosthesis rehabilitation and considering the advantages of digital workflows and monolithic materials, this study aimed to describe a therapeutic procedure and document the clinical results of implant-supported zirconia fixed partial prostheses (FPPs) with one cantilever extension replacing the missing lateral and posterior teeth.

## Methods

 We consecutively enrolled 15 patients who required dental implant rehabilitation with fixed dental prostheses. All the patients attended a private outpatient clinic from 2018 to 2021. The study was conducted in Italy, and all the procedures followed European laws and regulations.

###  The inclusion criteria

- Good health, according to the system of the American Society of Anesthesiology
- Aged > 18
- No general medical contraindications for implant therapy
- Two or more missing teeth from the canine to the second molar
- Good periodontal health or treated periodontitis

###  The exclusion criteria

- Smoking > 15 cigarettes a day
- Untreated periodontitis
- Pregnancy
- Acute infections
- Keratinized mucosal tissue < 2 mm.

A diagnostic wax-up of the missing teeth was performed on digital casts for each case. Each case was designed using CoDiagnostiX, Dental Wings software, to plan the implant position in the distal part of the edentulous ridge where the bone conditions were more favorable.The surgical procedures were performed according to the protocols recommended by manufacturers. The implant site was prepared after raising a full-thickness flap. Then, the implant was placed as designed in digital planning with the support of a surgical guide.

 The patients were recalled 3–6 months after surgery for a pre-prosthetic evaluation. A healing abutment was placed, and implant stability was proven.

 Three weeks after second-stage surgery, a definitive digital impression was taken using an intraoral scanner (TRIOS; 3Shape). Then, the prosthetic procedures were performed according to the recommendations of implant manufacturers.

All the prostheses were designed using CAD software (Ceramill Mind; Amann Girrbach AG) with a connector cross-sectional area of at least 12 mm^2^. Monolithic zirconia FPPs were fabricated using a CAD-CAM unit (Ceramill Motion 1; Amann Girrbach AG) from pre-sintered, pre-shade zirconia blanks (Ceramill Zolid HT + ; Amann Girrbach AG). The FPPs were characterized by ceramic-based colors (Initial IQ Lustre Paste; GC). The screw-retained zirconia FPPs were subsequently inserted, and a check of the occlusion was performed. The patients underwent a baseline examination after 1‒3 weeks of final prosthesis insertion. Aesthetics, proximal contacts, and occlusion were modified when needed following a careful examination.

**Figure 1:**
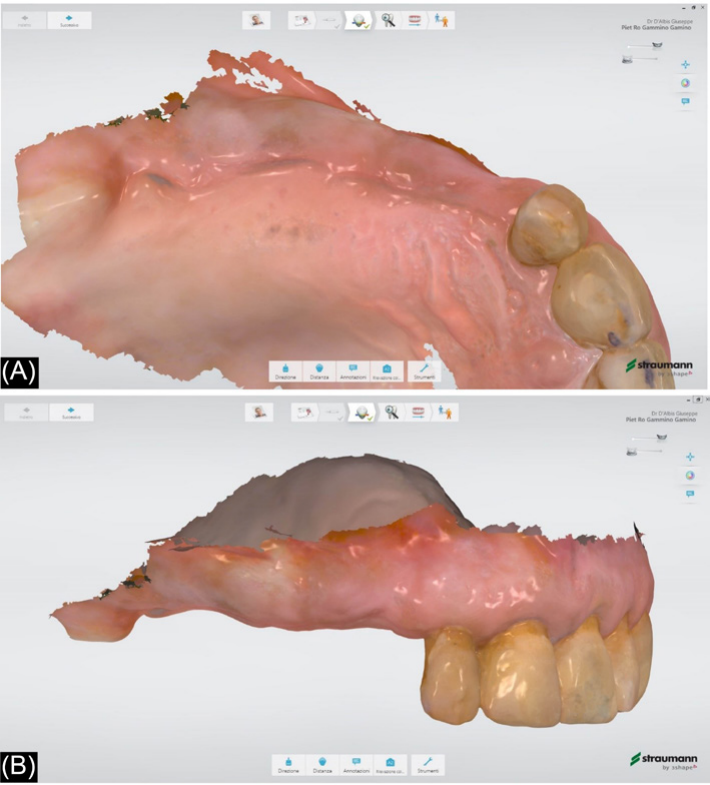
Initialdigital casts (A) Occlusal view (B) Buccal view

**Figure 2:**
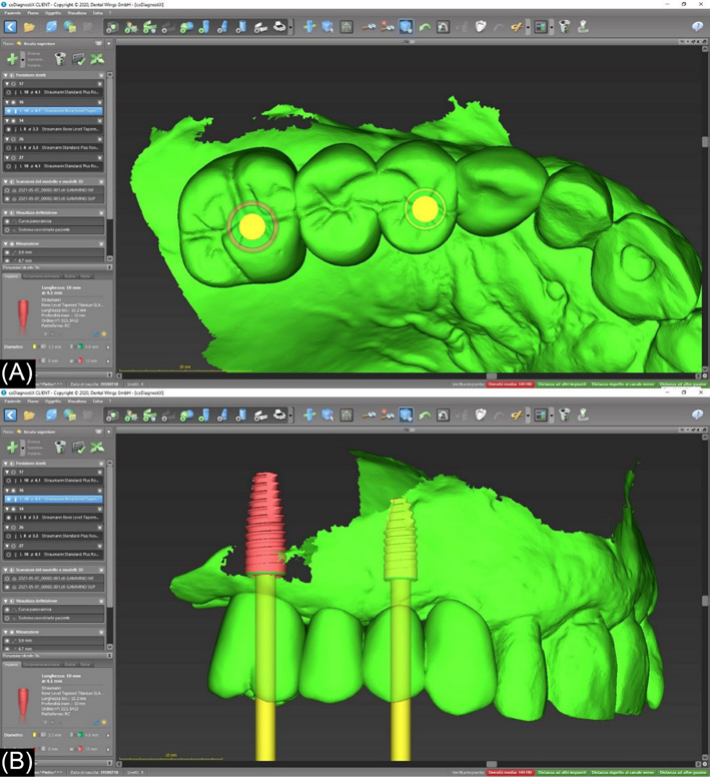
Planning of the implant positions in the CoDiagnostiX, Dental Wings software

**Figure 3:**
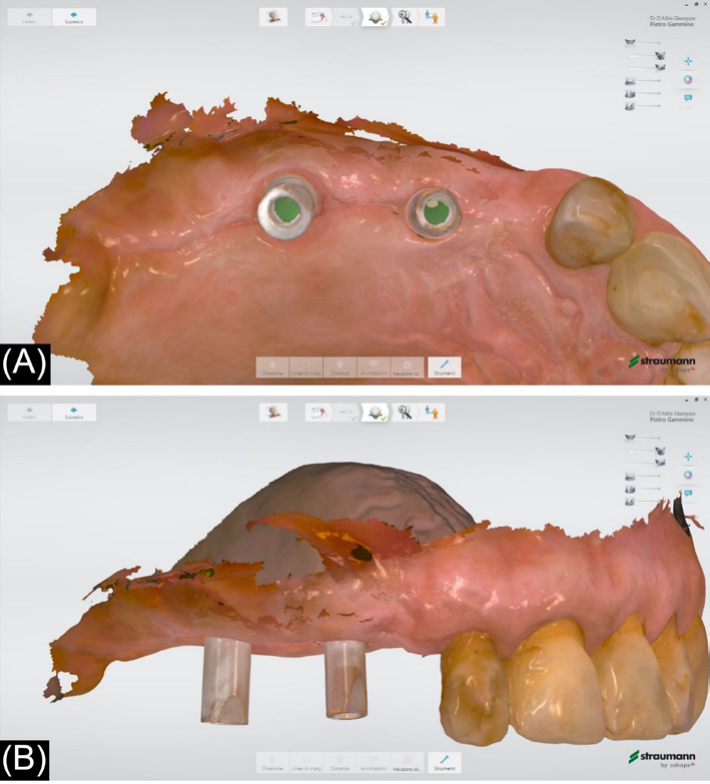
Definitive digital impression using an intraoral scanner (A) Occlusal view (B) Buccal view

**Figure 4:**
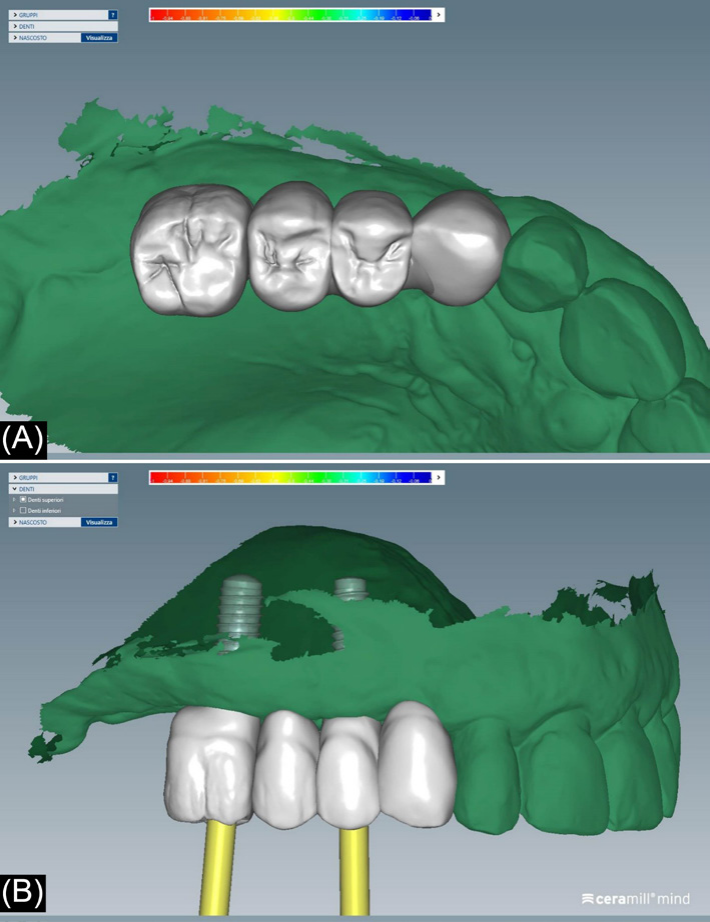
Designing of the prostheses using CAD software (A) Occlusal view (B) Buccal view

**Figure 5:**
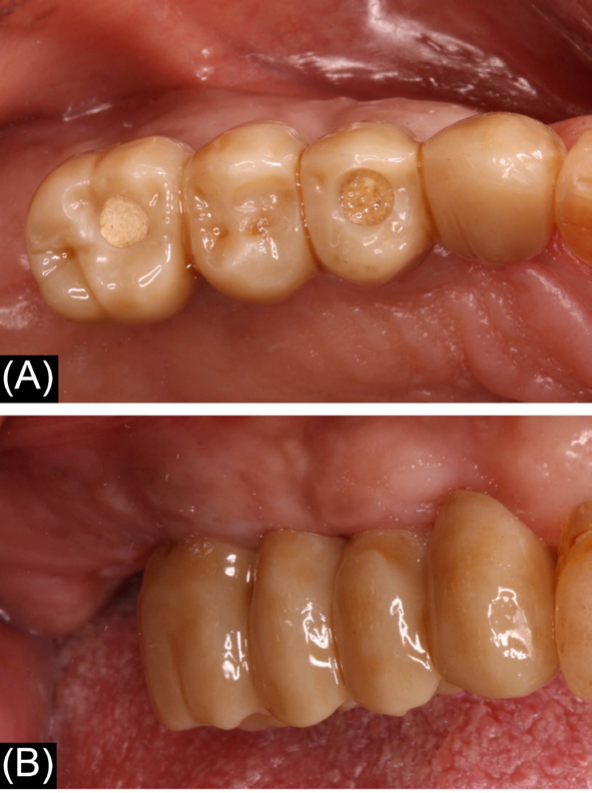
Clinical presentation of the Implant-supported zirconia fixed partial dentures (A) Occlusal view (B) Buccal view

**Figure 6:**
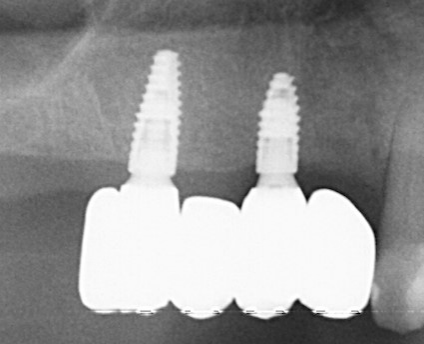
Periapical radiograph of the implant at two years follow up

The patients were recalled at least once a year to assess the restorations functionally and aesthetically, peri-implant tissues, and implant health status. An individual maintenance program with regular dental hygiene sessions was performed for every patient during the entire study period, and an x-ray was taken at the 2-year follow-up. Figures 1-6 show the workflow for one exemplary clinical case treated with one mesial cantilever implant-retainer FPP replacing missing lateral to posterior teeth.

## Results

Sixteen implant-supported cantilever zirconia FPPs inserted in 15 patients were observed for a mean period of 42 ± 6 months with a minimum of 36 months and a maximum of 48 months. There were eight female and seven male subjects with an age range of 41‒65 years (Table 1).

**Table 1:** Patient sample characteristics

**Characteristics**	
Patients treated	15
Patients followed	13
Mean age (SD)	57 (6)
Gender (M/F)	7/8
Mean number of bridges	1.09

SD, standard deviation.

 The age of the recruited patients ranged from 41 to 65 years old, with a mean age of 53 ± 12 years; 53% of the restorations were inserted in males, and 47% were inserted in females.

Thirty-four implants (20 Straumann, SLA Active; 14 Xive, Dentsply) supported 16 bridges, yielding a mean number of one FPP, two implants/patient, and two implants/bridge. Tables 2 to 4 present implant distributions in terms of the diameter, length, and tooth positions. The most common position for implants were the molars in the mandible and the first premolar and the first molar in the maxilla. Implants measuring 8 mm in length and 4.1 mm in diameter were the most frequently used ones.

**Table 2:** Implant locations according to their position in jaws

	**Implant position**							
	**1**	**2**	**3**	**4**	**5**	**6**	**7**	**Total**
Maxilla	-	-	-	4	3	5	1	13
Mandible	-	-	-	2	3	9	7	21

**Table 3:** Implant lengths in terms of jaws location

**Implant length (mm)**	**Maxilla**	**Mandible**	**Total (%)**
6	1	1	2 (5.9%)
8	6	4	10 (29.4%)
9.5	1	4	5 (14.7%)
10	3	5	8 (23.5%)
11	2	5	7 (20.6%)
13	-	2	2 (5.9%)
total	13	21	34 (100%)

**Table 4:** Implants diameters in terms of jaws location

**Implant diameter (mm)**	**Maxilla**	**Mandible**	**Total (%)**
3.3	4	1	5 (14.7%)
3.4	1	2	3 (8.8%)
3.8	-	5	5 (14.7%)
4.1	6	9	15 (44.1%)
4.5	1	4	5 (14.7%)
5.5	1	-	1 (2.9%)
Total	13	21	34 (100%)

 Despite the insertion of the implants in the location with the most favorable vertical and horizontal bone conditions, in two cases, a sinus lift was performed, and in two cases, the implants were tilted. In all the other cases, the implants were parallel, axial to the prosthetic axis.

 There were no peri-procedural and post-procedure complications, adverse events, or infections. In addition, no procedures resulted in swelling and hematoma of the surgical area.

Of the 16 FPPs followed, 12 were mesial cantilevered, and four were distal cantilevered. Natural teeth were antagonists for 11 cantilever FPPs and five contacted implant-supported prostheses (Table 5). Table 6 presents the number of the FPPs according to the cantilever location and number of implants.

**Table 5:** Details of fixed partial prostheses

**Characteristics**	
Number	16
Cantilever (mesial/distal)	(12/4)
Jaw(upper/lower)	(6/10)
Opposite dentition (teeth/crown)	(12/4)
Number of implants (range)	2 (1-3)
Mean number of teeth for FPP	3 (2-5)

**Table 6:** Distribution of cantilever locations in terms of the number of implants

**Number of implants**	**Mesial cantilever**	**Distal cantilever**
1	1	-
2	9	3
3	3	-

The distribution of cantilever location in the maxilla was as follows: canines 25% and premolars 18.7%. The distribution of cantilevers in the mandible was as follows: canines 6.3%, premolars 31.3%, and second molars 18.75% (Table 7).

**Table 7:** Distribution of cantilevers on the fixed partial prostheses

	**Cantilever position in the jaw**							
	**1**	**2**	**3**	**4**	**5**	**6**	**7**	**Total**
Maxilla	-	-	4	1	2	-	-	7
Mandible	-	-	1	1	4	-	3	9

 Implant complications occurred in 12.5% of the treated patients and 11.7% of the implants. Complications were registered for four implants in two patients presenting signs and symptoms of peri-implant disease two years after implant loading. Ultrasonic mechanical therapy and local antibiotics were provided. No further peri-implant alterations or bone loss progression were detected in follow-up visits.

 Bleeding on probing was followed up for 2.5 years in one patient with the previous peri-implantitis. No implant failure was observed. However, percussion with evidence of functional ankyloses was observed in all the implants.

 One case of FPP loosening of the abutment screw was observed in a partial prosthesis supported by one implant prosthesis, and the prosthesis was re-inserted. Within the observation time, no implant failure, prosthetic chipping, or fracture was noted, corresponding to a survival rate of 100%.

## Discussion

All complications were immediately resolved without any further complications. The presence of peri-implantitis in two cases was due to deteriorating general health in elderly patients. The biological complications were slightly lower than reported in a recent retrospective cohort study evaluating the outcomes of implant-supported metal-ceramic FPPs with cantilever extension; that study showed peri-implantitis in 26.9% of patients.^15^ The factors influencing this difference may have been differences in follow-up (in this study, there was little follow-up) and the fact that in the present study, all FPPs were screwed; in the other study, the FPPs were cemented. There is no scientific evidence that the material difference can minimize the possibility of peri-implantitis.

The prosthetic complication in one case was screw loosening in a single-implant-retainer FPP. In two previous studies on cantilevered single-implant-retainer metal-ceramic FPPs, most complications were screw loosening.^6,16^ In the present study, this technical complication may be related to using a single implant. There is no evidence that the rigidity of zirconia can influence screw loosening in FPPs.

 Using a cantilevered implant-supported restoration is a promising alternative in partial edentulous rehabilitations in economic and biological terms. Therefore, it can be considered a less invasive treatment approach that adheres to the concepts of prosthetically guided implantology. Making a treatment minimally invasive also means making it more pleasant for the patient by reducing the treatment time.

From this point of view, using digital workflows is substantially advantageous. The advantages include a positive patient experience of digital treatment, the precision and fit of the prosthetic product, and decreased occlusal and interproximal retouching that monolithic materials require.^17^

Contemporary studies point out the complications inherent in monolithic materials, particularly zirconia FPPs.^18,19^ However, the available data are sparse and conflicting due to diversities in the study designs.^20,21^

The posterior regions are subjected to significantly high occlusal forces ranging from 300 to 800 N.^22,23^ Due to the masticatory load, cantilevers show unfavorable biomechanical behaviors, primarily for distal cantilevers.^24^

Monolithic zirconia in this study yielded excellent clinical outcomes, including the ability to withstand masticatory forces without mechanical failure. The design of the zirconia framework increases the width of the connector area not less than 12 mm^2^.^25,26^ This design appears to be essential in terms of the biofunctional success of the posterior prosthesis primarily aimed for mastication as opposed to aesthetics.^27,28^ Even though a short cantilever was used (one tooth), the prosthetic structure resulted in a more favorable distribution of stress.^29^ This finding is undoubtedly an indispensable factor for the success of restorations.

 The positive outcome of implant-retainer monolithic zirconia cantilevers replacing posterior teeth relies on a digital prosthetic approach that must respect the length of the cantilever and the thickness of the material. Therefore, a careful study of the case is necessary, given the prosthetic structure that depends on the position of the implants.

 The survival rate of the inserted implant-retainer zirconia FPPs replacing molars, premolars, and canines was 100%. In addition, all the 16 followed prostheses were functionally and aesthetically successful.

 Given the promising performance of cantilevered implant-retainer zirconia FPPs replacing teeth in the lateral to the posterior area, further clinical trials are suggested to validate the reliability of these rehabilitations as an option for replacing missing lateral to posterior teeth.

## Conclusion

 This study suggests that implant-retainer zirconia cantilever FPPs provide a minimally invasive treatment with good clinical and patient-reported outcomes in replacing missing posterior teeth after four years in function.

## Funding

 None.

## Ethics Approval

 The study was conducted with national and international guidelines and registered with the identifier code NCT05526547.

## Competing Interests

 None.
